# Intractable Parathyromatosis despite extensive surgical interventions: A case report with literature review

**DOI:** 10.1016/j.ijscr.2023.109172

**Published:** 2023-12-15

**Authors:** Shada Khaled Bashantoof, Mansoor Abdulmajeed Alramadhan, Modhi Hamad Alawadh, Nada Abdulaziz Bin Samaih, Rania Abdullah Alshammari, Abdulsalam Aodah

**Affiliations:** a Riyadh 12788, Saudi Arabia; b King Saud Medical City, Riyadh 12745, Saudi Arabia; c King Khalid General Hospital, Hail 55211, Saudi Arabia

**Keywords:** Primary hyperparathyroidism, Parathyromatosis, Recurrence, Case report

## Abstract

**Introduction and importance:**

Parathyromatosis is a very rare condition where persistent hyperparathyroidism occurs after surgical excision of the parathyroid gland. That is explained by many theories mainly a seeding of parathyroid gland tissue post parathyroidectomy. We report a rare case presentation of Parathyromatosis and the clinical course with the consequences of her condition.

**Case presentation:**

a 38 years old female presented first time in 2003 with a parathyroid cyst that underwent excision which was ruptured accidentally and seeding of content happened. As a consequence, she had multiple presentations of recurrent hyperparathyroidism with different locations in the neck region in which parathyroid tissue was found invading muscle, subcutaneous tissue, and surrounding structure, she was managed surgically eight times during 20 years combined with medical management with intermittent remission.

**Discussion:**

Parathyromatosis diagnosis is challenging and requires differentiating it from other gland pathology such as adenoma or carcinoma, management must include meticulous en-block resection to eliminate all parathyroid tissue, recurrence is high which may require multiple surgeries.

**Conclusion:**

managing such a rare condition is very challenging in diagnosis and management ranging from medical therapy to Surgery which may reach multiple attempts to control the disease, we also refer to the reason possibly due to multiple theories of getting this condition. Such a long-term disease, it needs surveillance follow-up to prevent the complication and recurrence.

## Highlights

• Parathyromatosis is a highly rare condition that require multiple operations, no definitive treatment has been implied yet.
• Combination of medical and surgical intervention is used as a combination to achieve better control and symptoms relief.
• A differentiating between parathyroid carcinoma, adenoma, and Parathyromatosis is crucial in the management.
• Long term surveillance is advocated in such cases.

## 1 Introduction

Parathyromatosis is a rare condition defined as a recurrent manifestation of hyperfunction parathyroid gland. Many etiologies found in the works of literature might explain this condition, summed up in mostly improper surgical technique that leads to a rupture and seeding of parathyroid tissues, which could be noticed during or after the operation, another theory mentioned the possibility of implantation of genetically mutated parathyroid gland that turned to be hyper functioned, some cases they return the causes to parathyroid malignancy [1]. We found in the literature that it's a very complicated condition and challenging management that require a surgical intervention as a standard of care and a medical management as supplementary to control the disease nature. Most of the cases suffering from this condition will frequently manifest with osteoporosis and renal stones. We are reporting here a case about persistent Parathyromatosis and our challenges in the management of this rare case in our community medical city. The work has been reported in line with the SCARE criteria [14].

## 2 Case

**Fig. 1:**
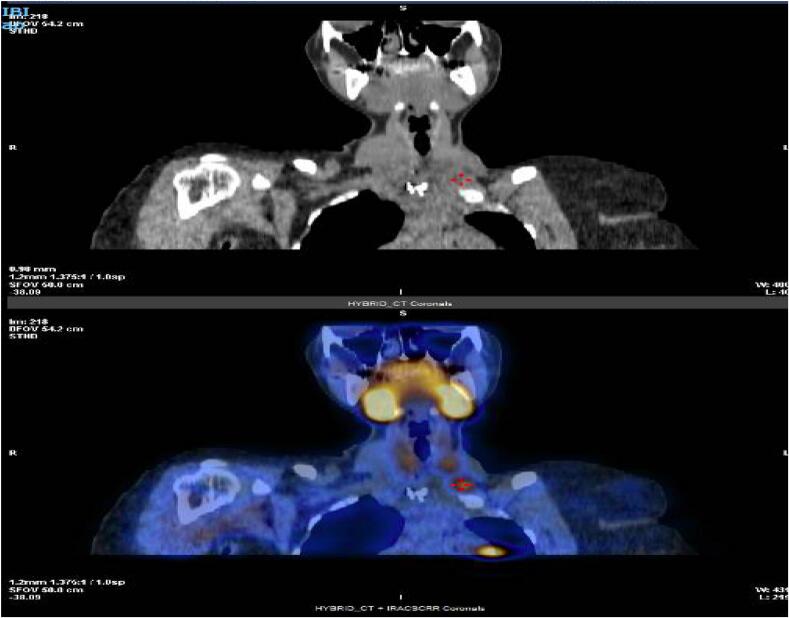
SPECT revealed hyperactivity of parathyroid tissue.

**Fig. 2:**
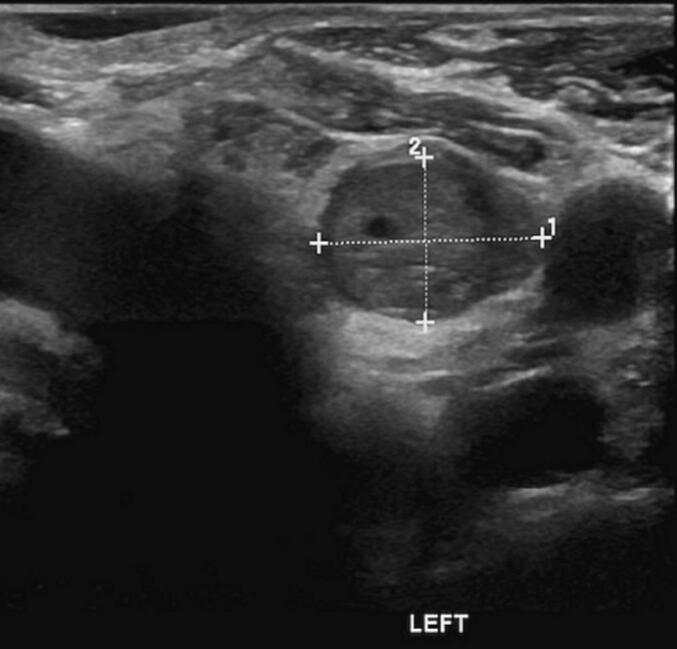
Left parathyroid adenoma measuring 1 cm.

**Fig. 3:**
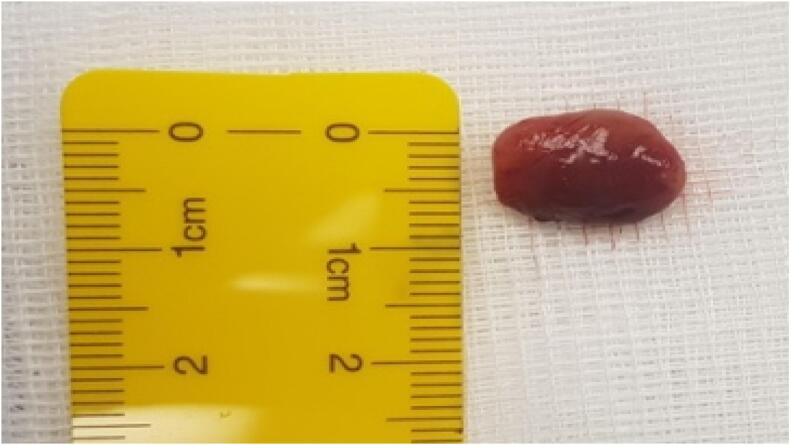
parathyroid nodule.

38 years old female operated eight times for the last twenty years for recurrent primary hyperparathyroidism. The first operation was done in 2003 at the age of 18, the patient presented initially to the emergency department with lower limbs pain. The laboratory results showed high serum parathyroid hormone (PTH) level of 3552 pg/ml (Normal range: 10–60 pg/ml) and alkaline phosphatase (ALP) level of 2920 mg/dl (normal range 50-150 mg/dl). Neck ultrasound (US) showed a single 4 × 3 cm cystic area left to the thyroid region. (99 m)Tc-MIBI scan revealed abnormal uptake in the upper mediastinum with normal thyroid. She underwent excision of the cyst with accidental rupture of the functioning parathyroid cyst resulting in spillage of the contents in the operative field, discharge safely with normal calcium level at that time. In 2006 she was admitted with a history of stable neck swelling for 1 year. Serum calcium was slightly elevated at 2.82 mmol/L (Normal rang 2.0–2.52 mmol/L), PTH 224 pg/ml, ALP 155 mg/dl. Neck US and (99 m)Tc-MIBI scan showed parathyroid adenoma 1.5 cm anterior to left sternocleidomastoid. Excision of the nodule was done, and intra-op PTH dropped from 573 to 206 pg/ml. Histopathology examination revealed parathyroid adenoma. By 2008, she had recurrent bone pain. Investigated by Computed tomography (CT) and (99 m) Tc-MIBI scan with findings of right inferior parathyroid lesion. Neck exploration and excision of the parathyroid gland implanted in sternocleidomastoid and subcutaneous nodules were performed. Intraoperative PTH exhibits a non-significant drop. Post-operative (99 m) Tc-MIBI scan revealed normal region uptake and serum calcium level was in the high upper range. In 2012, she had classical symptoms of hypercalcemia. Parathyroid hormone was high at 2204 pg/ml. Multiple well-defined, midline, subcutaneous, infra-hyoid soft tissue lesions largest was 15x12mm in the anterior border of the right sternomastoid were detected in US and MRI neck. Neck exploration was done with complete excision, and intraoperative PTH significantly dropped. Two years later, she was found to have nephrolithiasis and palpable nodules in the previous collar scar along with a high PTH level of 386.6 pg/ml and calcium level of 3.05 mmol/L. (99 m) Tc-MIBI scan showed right inferior parathyroid adenoma. Excision of hypertrophic collar incision scar with extensive dissection over left sternocleidomastoid muscle and excision of parathyroid subcutaneous implantation was done. Histopathology revealed multiple parathyroid nodules with cystic change. After six months of follow-up, PTH gradually increase to 179.5 pg/ml, and a soft tissue lesion, located left side of the midline of the neck root area, about 10 mm x6mm was found in the US, and planned for follow-up at that time. In 2017, (99 m) Tc-MIBI scan was repeated with the same findings of parathyroid adenoma, calcium level fluctuating 2.7–2.9 mmol/L, started on cinacalcet and Cholecalciferol. Despite medical management, she had persistent hyperparathyroidism. US neck and SPECT-Mibi scan has suggestive of left inferior parathyroid adenoma with another focal uptake in right muscular region (Fig. 1, Fig. 2). In 2018, surgical intervention was decided, and the patient underwent left inferior parathyroidectomy and right subcutaneous cyst excision, PTH dropped from 1003 to 545.9 pg/ml. The patient's bone pain had reoccurred, and there was ectopic parathyroid at the lower part of the left sternocleidomastoid muscle seen in the neck ultrasound. Medical therapy continued with the addition of Denosumab. later on, excision and dissection of the left parathyroid deposit in the left sternocleidomastoid muscle were done with a significant drop in PTH. In 2020, she was found to have high PTH 323 pg/ml after her follow-up, and (99 m) Tc-MIBI demonstrated a new parathyroid recurrence above the superior pole of the left lobe and ectopic parathyroid adenoma in the left sternomastoid location (Fig. 3). Underwent excision of 3 ectopic parathyroid nodules with significant PTH drop. Over the last 2 years, she has been followed up and kept in medical therapy, with a current good control in serum calcium level. The case is summarized in Table 1.

**Table 1:** Case report timeline.

Year	Presentation	Procedure	IntraOp PTH	Histopathology
2002	**Lower limb pain for 6 months**^a^	**Excision of the cyst with accidental rupture**	**None**	
2006	**Constant neck swelling for 1 year**	**Excision of the neck nodule** **Under local anesthesia**	**573 > 206** pg/ml	Parathyroid adenoma
2008	**Recurrent bones pain**	**Left parathyroidectomy and Excision of the subcutaneous nodule**	**370 -** **> 342** pg/ml	parathyroid tissue implanted in the platysma muscle
2012	**Recurrent bones pain and limbing + renal stones**	**Neck exploration - excision of parathyroid tissue**	**1054 -** **> 142** pg/ml	**Parathyroid cells** inf**iltrated sternomastoid, subcutaneous tissue and the mass in the** sup**ra sternal tissue**
2014	**Multiple nodules at previous collar scar**	Excision of the hypertrophic collar incision scar with extensive dissection over the left sternocleidomastoid muscle and excision of the parathyroid subcutaneous implantation	**386 > 123.5** pg/ml	Multiple parathyroid nodules with cystic change
2018	**Bilateral renal stones + bone pain**	Left inferior parathyroidectomy and right subcutenious cyst excision	1003 > 545.9 pg/ml	
2018	**Bilateral renal stones + bone pain**	Execion and dissection of a left parathyroid deposit	503.5 > 152 pg/ml	
2020	**Bilateral renal small stones**	Excision of 3 ectopic parathyroid nodules	**475 > 190** pg/ml	

a She presented with bilateral vuge bone pain in which labs invistigations showed high calcium level

## 3 Discussion

**Fig. 4:**
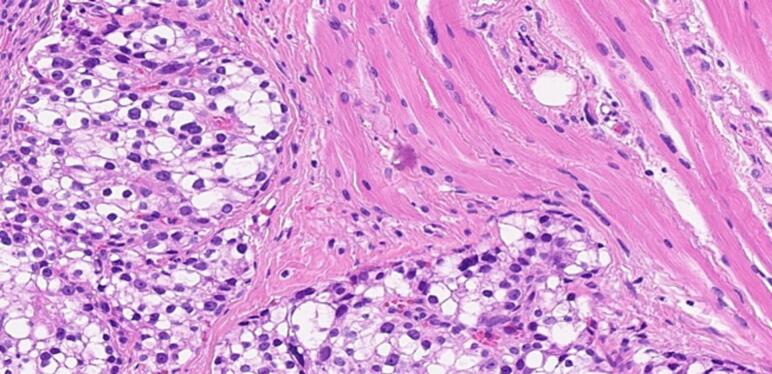
The adjacent skeletal muscle fibers are invaded by the parathyroid tissue.

**Fig. 5:**
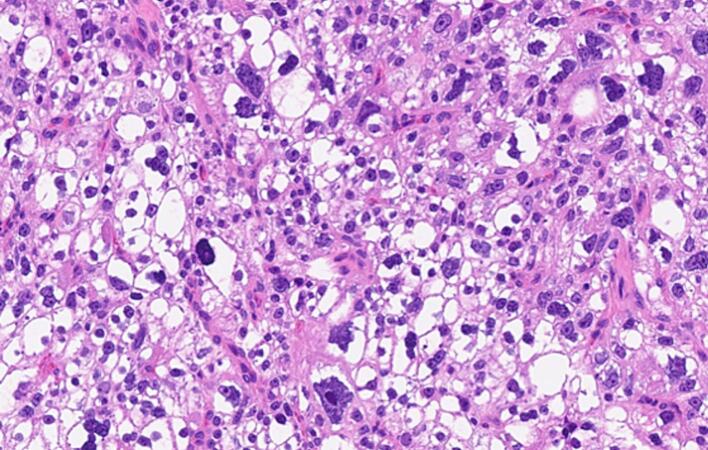
The atypical nuclear features including nuclear pleomorphism, visible nucleoli and coarse chromatin, are demonstrated.

**Fig. 6:**
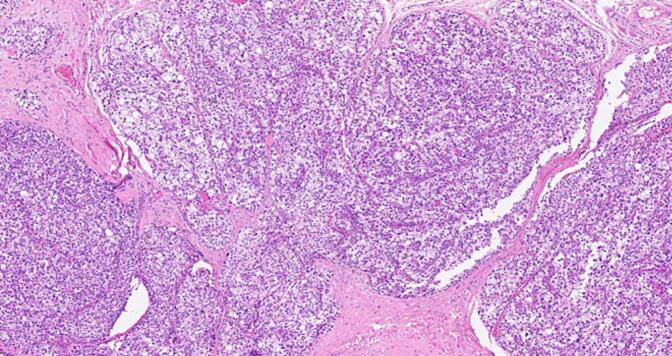
Fibrous bands traversing the parathyroid tissue are seen in this picture.

The incidence of primary hyperparathyroidism is fluctuating from 34 to 120 per 100,000 person-years in a study of 15,234 patients with chronic hypercalcemia [2]. As such, 85 % of them had single adenoma as a primary cause while <1 % had parathyroid cancer [3], Parathyromatosis is rare, and the incident percentage had not been acknowledged till this century. The first case was reported by Palmer et al. 1975 [4], up to our knowledge of the literature review indexed in PubMed, only twenty-eight cases of Parathyromatosis due to primary hyperparathyroidism were reported [5], summarized in Table 2. More than one theory had been conducted to identify the possible causes. In 1977 Reddick et al. [6] described 2 types of Parathyromatosis: type 1 is defined as spillage and subsequent seeding of parathyroid adenomas or cysts, after parathyroidectomy, type 2 usually due to embryonic development of parathyroid tissue nests under physiological condition. Another uncommon possibility had been presented by Sim et al., a case of Parathyromatosis due to spontaneous adenoma rupture prior to presentation [7]. Parathyroid adenoma and carcinoma are crucial deferential diagnoses as the management will be affected, both present with hypercalcemia. Histologically it's challenging to differentiate between them. According to the 5th edition of the WHO Classification of Tumors, Endocrine and neuroendocrine tumors the locally infiltrative growth of Parathyromatosis may be indistinguishable from parathyroid carcinoma histologically; however, biomarker studies, markedly elevated serum calcium and PTH levels, palpable neck mass, and more importantly presence of vascular or perineural invasion can help distinguish parathyroid carcinoma [8].Although microscopically we identified some atypical features in our case including nuclear atypia with a high N/C ratio, pleomorphism, prominent nucleoli, and the presence of fibrous septa with skeletal muscle invasion\pseudoinvasion, we acknowledged the absence of lymphovascular and/or perineural invasion (Fig. 4, Fig. 5, Fig. 6). Based on the same reference, in the clinical context of our case, the appropriate diagnosis would be Parathyromatosis rather than carcinoma.Lab investigations and localization imaging studies cannot differentiate between these major entities. A meta-analysis demonstrated that ultrasound and Sestamibi-SPECT have comparable and similar accuracy at 76.1 % vs 78.9 %, while 4D-CT remains superior in accuracy at 89.4 % [9]. Management is contributed to the most challenging part with high anticipation of recurrence is kept in mind, therefore a meticulous resection with high experience endocrine surgeon is mandatory. The initial management in such cases is surgical en block resection, routine cervical thymectomy is sometimes indicated in both types due to the high rate of ectopic parathyroid glands [7]. Patients who are not a candidate for surgery are managed medically by a combination of calcimimetic and bisphosphonate medications as described by Scorza et al [10] Nakai et al., conducted a study to review clinical guidelines for the management of secondary hyperparathyroidism in CKD patients, comparing the effectiveness of combined therapy including calcimimetics and vitamin D analogs to improve the outcome of mineral and bone disorders. However, it showed that cinacalcet is extremely effective in recurrent hyperparathyroidism due to Parathyromatosis as it inhibits the proliferation of parathyroid cells as well as its action on calcium-sensing receptors of parathyroid glands, it also may cause apoptosis of parathyroid tissue if giving in a high dose [11]. Co-administration of paricalcitol and ibandronate showed a good response as reported by Daphnis et al [12] Our case was started on a trial of medical management after the 4th reoperation, which was not effective and did not control her condition, as she still had persistent hyperparathyroidism and went for four more reoperations. The rate of recurrence is high, parathyroid tissue could not be seen during the operation or resected completely as the locations are not anticipated, Sudha et al. surprisingly reported tissue seeding in the endoscopic operation tracts [13]. Most of the reported cases were reoperated a few times unlike our case which had multiple flares of the disease that required 8 extensive surgical interventions, she showed partial control that required medical management over the years.

**Table 2:** Case reports from 1977 to 2023.

	Author	Cases	Primary dx	Medical treatment	Surgical treatment	Outcome
1	Reddick et al.,1977	1	PT hyperplasia	–	2 reoperations	Remission
2	Rattner et al.,1985	4	Lt inf. PT cystic adenoma – Rt inf. PT adenoma – hyperplasia – Rt inf adenoma	–	1–3 - 1–3 reoperations	Remission
3	Akerstrom et al.,1988	3	Primary hyperparathyroidism	–	1/2/2 reoperation	Remission
4	Sokol et al., 1993	1	Primary hyperparathyroidism	–	2 reoperations	Persistent
5	Evans et al., 2005	1	Rt PT adenoma	–	1 reoperation	Remission
6	Tublin et al., 2007	1	Rt PT adenoma	–	3 reoperations	Remission
7	Diaconescu et al., 2011 ^(24)^	1	Rt inferior PT adenoma	–	1 rexcision + thyroid lobectomy	Remission
8	Mohammadi et al., 2012	1	Lt & Rt inf. PT adenomas	–	1 reexcision	Remission
9	Hage et al.,2012	1	hyperplasia of right lower gland +n right mediastinal adenoma	Bisphosphonate + Cinacalcet	4 reoperations	Mild remmission
10	Twigt et al., 2013	2	Rt inf. Parathyroid adenoma – retrosternal/paratrachial adenoma	–	1 and 3 reoperations	Remission
11	Pinnamaneni et al., 2013	1	hyperparathyroidism	–	2 reoperations	Persistent
12	Sim et al., 2013	1	Rt superior parathyroid adenoma	Cinacalcet	10 reoperation	Remission
13	Scorza et al., 2014	1	Lt inf. PT adenoma	Cinacalcet + alendronate	3 reoperations	Persistent
14	Edling et al., 2014	1	P HPT	cinacalcet	1 reoperation	Remission
15	Sharma et al., 2016	1	Lt inf. Parathyroid adenoma	–	2 reoperations	Persistent
16	Jain et al., 2016	1	Rt inf. Parathyroid adenoma	Calcimimetic and cinacalcet Bisphosphonate and alendronate	2 reoperations	Remission with medical therapy
17	Aggarwal et al., 2017	1	Lt sup. Parathyroid adenoma	–	1 reoperation	Remission
18	Trevor et al., 2018	1	Rt Parathyroid adenoma, Mediastinal cyst	–	2 reoperations	Remission
19	Haciyanli et al., 2019	1	Lt sup. Parathyroid adenoma	–	2 reoperations	Remission
20	Abraham et al., 2019	1	Rt sup. Parathyroid adenoma	Cinacalcet	1 reoperation	Remission with medical management post op
21	O altin et al., 2020	1	Retrosternum & pericardial adipose tissue parathyroid tissue	–	1 operation	Remission
22	Elena et al., 2021	1	Rt sup. Parathyroid adenoma	Cholecalciferol	1 reoperation	Remission

Table 2: number of Parathyromatosis cases due to primary cause since 1977.

## 4 Conclusion

Parathyromatosis can become refractory to surgical and pharmacologic treatments, requiring multiple interventions. Careful and complete resection of intact parathyroid tissue is key for successful treatment. Patient education regarding the possibility of recurrence is imperative to ensure compliance with follow-up.

## Consent

Written informed consent was obtained from the patient for publication and any accompanying images. A copy of the written consent is available for review by the Editor-in-Chief of this journal on request.

## Ethical approval

This case study was approved by the institutional research and innovation center at king saud medical city (H2RI-31-May23-01).

## Funding

This research did not receive any specific grant from funding agencies in the public, commercial, or not-for-profit sectors.

## Author contribution

Shada B. devised the project, the main conceptual ideas, and proof outline as well as writing the discussion and modulation of the manuscript, Modi A. contributed to writing the case and data collection, Nada S. contributed by writing the introduction and abstract, Rania A. contributed in references writing and arrangement, Mansoor A. helped in supervision of the manuscript and modulate it, final version and provided critical feedbacks, Abdulsalam A. helped in writing the histopathology part. Data collection is done by: Shada K., Modi H., Nada S.

## Guarantor

Dr. Mansoor Abdulmajeed Alramadhan.

## Declaration of competing interest

The authors have no conflict of interests to declare.
